# The Trail Runners’ Tendon—How Do Weekly Mileage and Elevation Gain Affect Achilles and Patellar Tendon Morphology?

**DOI:** 10.3390/jfmk10010001

**Published:** 2024-12-24

**Authors:** Alberto Rubio-Peirotén, Antonio Cartón-Llorente, Hendrik Mugele, Diego Jaén-Carrillo

**Affiliations:** 1 Faculty of Health Sciences, Universidad San Jorge, 50830 Zaragoza, Spain; arubio@usj.es; 2 Department of Sport Science, Universität Innsbruck, 6020 Innsbruck, Austria; hendrik.mugele@uibk.ac.at (H.M.); diego.jaen@uibk.ac.at (D.J.-C.)

**Keywords:** trail running, Achilles tendon, ultrasonography, accumulated elevation, cross-sectional area

## Abstract

**Background**: Unlike road running, mountain and trail running typically cover longer distances and include uphill and downhill segments that impose unique physiological and mechanical demands on athletes. **Objectives**: This study aimed to identify morphological differences in the patellar and Achilles tendons between trail and road runners. Moreover, the potential influence of weekly mileage and accumulated positive elevation gain on the morphology of both tendons was obtained. **Design**: Cross-sectional comparative study. Methods: Thirty-three road runners (11 women, 22 men) and thirty-three trail runners (13 women, 20 men) were recruited and their weekly mileage and elevation gain collected. All participants had a weekly training volume exceeding 20 km. The thickness and cross-sectional area (CSA) of their patellar and Achilles tendons were evaluated using ultrasound. **Results**: Independent samples *t*-tests revealed significant differences between groups for the Achilles tendon (*p* < 0.003) but not for the patellar tendon (*p* > 0.330). Further, Spearman’s correlation coefficients indicated moderate positive correlations for the thickness and CSA of the Achilles tendon with weekly running volume (0.256 and 0.291, respectively) and with elevation gain (0.332 and 0.334, respectively), suggesting a tendency for the tendon to adapt to greater training loads, enhancing its structural integrity and resilience. **Conclusions**: Trail runners exhibit larger and thicker Achilles tendons, likely due to increased weekly mileage and elevation gain, highlighting the adaptive response to mechanical overload from uphill running.

## 1. Introduction

Running is one of the most widely practiced sports globally [1]. In particular, trail running has seen a surge in popularity, with the number of participants doubling over the past decade [2]. Trail running events include not more than 20% of running on even asphalted roads, with the remainder being unpaved, uneven terrain [2], and thus, it possesses unique physiological and biomechanical characteristics [3]. However, despite its growing popularity, these characteristics have not yet been extensively studied.

The biomechanics of trail running cannot be fully understood without analyzing the behavior of the musculoskeletal system, particularly tendons. During running, lower-limb tendons play a critical role in the stretch–shortening cycle [4], which allows the tendons to store and release the energy necessary for efficient running mechanics [5]. Trail running, characterized by elevation changes in the terrain, affects running and thus alters the stress placed on the lower-limb tendons compared to road running. Research has shown that runners tend to adopt a mid-foot or forefoot strike pattern when running uphill [6,7], which places increased demands on the Achilles tendon (AT) and ankle [8]. During uphill running, the greater involvement of the Achilles tendon is due to a pre-stretch condition during the run cycle, which necessarily results in increased stress on posterior chain tendons and muscles. Conversely, when running downhill, most runners shift to a rearfoot strike pattern [9], which places more strain on the patellar tendon (PT) and quadriceps muscles due to a greater range of knee flexion and increased eccentric braking during landings, thereby contributing to overloading these structures [10]. In this regard, changes in running technique, such as those mentioned in the initial contact pattern, have been identified as key contributing factors to running-related injuries [11].

The main lower-limb tendons of road runners (PT and AT) have been extensively studied [12,13,14,15]. A key finding in the literature is that runners tend to have larger tendons, both in terms of thickness and cross-sectional area (CSA), compared to untrained or sedentary individuals [12,13,14,15]. These morphological differences are thought to result from the greater mechanical load experienced by runners’ tendons [16], prompting an adaptive response in the form of increased collagen production and extracellular matrix proliferation by tenocytes [17]. As a result, hypertrophy and increased stiffness have been observed in runners’ tendons [18], which, in turn, may provide a protective effect for muscle–tendon units. In this context, the so-called repeated bout effect evokes the ability of the musculotendinous system to trigger an inherent protective mechanism in response to damage caused by eccentric exercise, initiating an adaptive process that enhances resistance to future damage [19]. Furthermore, adaptations in runners’ tendons have been shown to reduce the metabolic cost of running [20] and enhance performance in distance running [21].

The morphological characteristics of trail runners have been less studied compared to those of road runners. One of the few studies investigating this found no significant differences in AT morphology between trail and road runners [22]. Of note, weekly running distance was nearly identical between both groups [22], and accumulated elevation was not considered. Given the recent exponential growth in trail running, a more detailed investigation of the main tendons of the lower limbs in trail runners is warranted.

Tendon morphology has been shown to influence various biomechanical variables, such as lower limb stiffness or duty factor [23,24,25]. Therefore, examining the tendon morphology of trail runners could provide valuable insights into their running biomechanics and potentially enhance their performance. Additionally, tendinopathies are a common injury among trail runners [26], highlighting the need to investigate how sport-specific intrinsic factors, such as weekly training volume and elevation gain, impact tendon morphology.

Given the established link between tendon morphology and running biomechanics [16], further research is needed to assess the morphology of the main lower limb tendons in trail runners, taking into account intrinsic factors specific to trail running, such as accumulated elevation or the weekly running volume. The present study has two main objectives: (i) to analyze the morphological differences in AT and PT between road and trail runners, focusing on thickness and CSA; and (ii) to investigate how weekly running volume and accumulated elevation gain influence potential tendon morphology differences. We hypothesize that trail runners, due to higher running volume and greater accumulated elevation gain, will exhibit tendons with increased thickness and CSA compared to road runners.

## 2. Materials and Methods

### 2.1. Subjects

Thirty-three experienced road (11 women, 22 men) and thirty-three trail runners (13 women, 20 men) participated in this study. The inclusion criteria were (i) being 18 years of age or older; (ii) running at least three times per week and covering a minimum of 20 km weekly; (iii) having competed in at least two running events per year over the past two years; and (iv) no lower limb tendon-related injuries (e.g., tendinopathies) in the six months prior to data collection. An a priori power analysis [17] was performed to determine the required sample size based on an assumed large effect size (d = 0.8), an alpha level of 0.05, and a desired statistical power of 0.80. The analysis indicated that a minimum of 26 participants per group would be needed to reliably detect significant differences in tendon morphology. To safeguard against potential dropouts due to injuries or scheduling conflicts with competitions, the study recruited a total of 33 runners for each group, ensuring the robustness and validity of the results. Recruitment was conducted using convenience and snowball non-probability sampling. Each participant received a thorough verbal and written explanation of the study’s objectives, methods, and potential risks. Subsequently, all provided written informed consent, in line with the ethical standards set by the World Medical Association’s Declaration of Helsinki. The research protocol was approved by the local Ethics Committee (No. 36/2023).

### 2.2. Material and Testing

For descriptive purposes, participants’ height (cm) and body mass (kg) were determined using a precision stadiometer and a weighing scale (SECA 222 and 634, respectively, SECA Corp., Hamburg, Germany). Additionally, data on the weekly running volume (in km) and the accumulated elevation gain (in m) were collected from each participant (Table 1). Of note, elevation gain refers to the total accumulated positive elevation, and it is important to stress that the negative elevation change will be equivalent in magnitude, as downhill running is inherently included in this parameter. Therefore, by mentioning elevation gain, we implicitly account for the work done during the descent as well.

**Figure 1:**
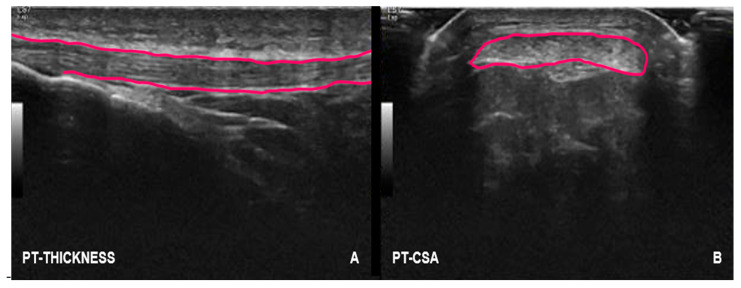
Ultrasound images of PT (i.e., thickness (**A**) and CSA (**B**)). Thickness and CSA are highlighted in pink.

The assessment of tendon morphological characteristics was carried out using high-definition ultrasound images (longitudinal and transversal views) acquired in B-mode using a wireless 12 MHz linear array probe (GE VScan Air CL, Freiburg, Baden-Wurttemberg, Germany) and with a gain of 100 dB. Each measurement was performed twice by a highly experienced researcher with over a decade of expertise in diagnostic ultrasound imaging. The clearest image, as determined by the examiner, was selected for subsequent calculation of morphological variables. To assess thickness and CSA, the ImageJ software (version 1.54k, NIH, Baltimore, MD, USA) was used [27]. The polygon tool within the software was applied to compute CSA. Given the known correlation between body mass and tendon morphology [28], CSA and thickness values were also adjusted to one-third of the participant’s body mass for the statistical analysis [14]. The PT characterization was evaluated with runners in the supine position, with both knees 30° bent [24,27]. This position was controlled and guaranteed throughout the examination. A reference of 1 cm distal to the lower pole of the patella, identified by the ultrasound device, was used to assess the tendon thickness and CSA [24,27] (Figure 1).

**Figure 2:**
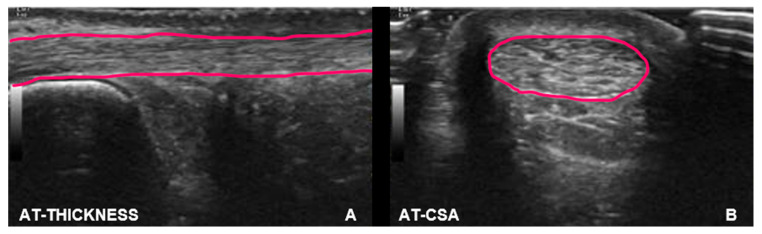
Ultrasound images of AT (i.e., thickness (**A**) and CSA (**B**)). Thickness and CSA are highlighted in pink.

During the evaluation of the AT, runners were in a prone position with both knees extended and their feet positioned outside of the bed, keeping the ankle in neutral position [25,27]. This position was controlled and guaranteed throughout the examination. Thickness and CSA were measured 3 cm proximally to the insertion into the calcaneus, measured using the ultrasound device [25,27] (Figure 2).

### 2.3. Statistical Analysis

Statistical analysis was performed using the Jamovi software package (version 2.3.26, The Jamovi Project). The normality of the variables was evaluated using the Shapiro–Wilk test. For variables that met the assumption of normality, a Student’s *t*-test for independent samples was applied to assess differences in AT and PT morphology between road and trail runners. When the assumption of normality was violated, the Mann–Whitney U test was applied, with effect sizes expressed as rank-biserial correlations [28]. Effect sizes were calculated and interpreted following Cohen’s criteria, where values of 0.2, 0.5, and ≥0.8 represented small, medium, and large effects, respectively [29]. Pearson’s correlation coefficients were calculated to evaluate the relationship between weekly running volume, elevation gain, and tendon morphology. Partial correlation analyses were conducted to control for the potential confounding effects of elevation gain on weekly running volume and vice versa, providing a clearer understanding of the independent relationships between each variable and tendon morphology. Additionally, Spearman’s correlation coefficients were used to assess the associations between weekly volume, elevation gain, and tendon morphology in the absence of normality. The statistical significance was set at *p* < 0.05.

## 3. Results

### 3.1. Patellar and Achilles Tendon Differences Between Road and Trail Runners

Both absolute and relative PT thickness and CSA showed no significant differences (*p* > 0.330) between road and trail runners (Table 2).

For the AT, significant differences were observed in both absolute and relative thickness, and for CSA, significant differences were observed between road and trail runners (*p* < 0.001; Table 2).

### 3.2. Influence of Weekly Volume and Elevation Gain on Patellar and Achilles Tendons

No significant correlation between weekly training volume and elevation gain on PT thickness and CSA, in absolute or relative values, was found. Also, when controlled for elevation gain, weekly training volume did not correlate with any PT morphology variable. Similarly, when controlling for the heterogeneous weekly training volume between road and trail runners, no significant associations were found for the PT (Table 3).

There was a significant correlation between weekly training volume and elevation gain on AT thickness and CSA in absolute and relative values (all, r > 0.327, *p* < 0.004). However, when controlling for elevation gain, a significant association between weekly running volume and both the absolute thickness (Spearman’s ρ = 0.256; *p* = 0.040) and CSA of the AT (Spearman’s ρ = 0.291; *p* = 0.019) was found. There was also a significant association between elevation gain and the CSA of the AT, both absolute (Spearman’s ρ = 0.332; *p* = 0.007) and relative (Spearman’s ρ = 0.334; *p* = 0.007). In contrast, no significant associations were detected for the AT thickness (Table 3)

## 4. Discussion

The aim of this study was twofold. On the one hand, to analyze the morphological differences of the AT and PT between road and trail runners, and on the other hand, to investigate how weekly running volume and accumulated elevation gain influence potential tendon morphology differences. In accordance with our initial hypothesis, the results show that trail runners present larger AT but similar PT than road runners. In addition, weekly running volume and accumulated elevation gain positively correlate with the CSA of the AT.

When comparing the morphological differences between the AT and PT of road and trail runners, it was observed that while both CSA and thickness of the AT were significantly larger in trail runners, in the case of the PT, there were no significant differences. Regarding the morphologic tendon differences between road and trail runners, there is not much evidence. To the best of the authors’ knowledge, only one study has previously investigated the morphological differences in the AT between road and trail runners [17]. Contrary to our results, Dar et al. [22] did not find significant differences between both types of runners either in the thickness or in the CSA of the AT. This discrepancy between these findings is initially easily attributable to the weekly running volume. While in the study by Dar et al. [22], the weekly running volume was very similar between road and trail runners, in our study, the weekly running volume was significantly higher for trail runners. This difference in weekly running volume is not an unimportant aspect since the load that the tendon is demanded of in relation to the weekly running volume is one of the main factors that explain the morphological adaptations found in the runners’ tendons [16]. Of note, a prior investigation assessed elite road and trail runners regarding their running economy, strength, and power [3]. The findings indicated that trail runners exhibited superior maximal power compared to road runners when running on flat terrain, although their cost of running also increased. The researchers suggest that the increased strength and power observed in trail runners may be due to the intense uphill and downhill training they perform, which, in turn, results in a more force-oriented strength–speed profile and jeopardizes their running economy compared to road runners. This explanation would justify the differences in CSA and thickness found in our study as tendon structural adaptations in response to chronic overloaded running.

Previous studies have reported how morphological differences in the AT and PT between runners and untrained subjects are mainly attributable to the greater tendon demand involved in running [12,13,14,15,30,31]. Furthermore, these tendon adaptations in response to loading seem to be related not so much to the magnitude of the load but to the volume since the differences mentioned above occurred in long-distance runners but not in sprinters [32,33]. The significant positive correlations for both AT thickness (r = 0.270) and CSA (Spearman’s ρ = 0.291) with weekly running volume suggest that higher training volumes are associated with increased tendon dimensions, reflecting a tendency for the tendon to adapt to greater training loads, and potentially enhancing its structural integrity and resilience.

A key characteristic of trail running is the weekly elevation gain. However, the influence of such an accumulation of vertical meters per week on the lower limb tendon morphology has received little attention in the past. To the best of the authors’ knowledge, this is the first study where this relationship has been assessed. For elevation gain, the significant associations with CSA and thickness for the AT highlight its importance in relation to tendon morphology. Moderate correlations (Spearman’s ρ = 0.332 for CSA and Spearman’s ρ = 0.334 for relative thickness) indicate that as elevation gain increases, the tendon’s CSA and the relative thickness also tend to increase, which may suggest adaptive responses to the biomechanical demands of varied terrains, highlighting the crucial influence of elevation gain on AT morphology. However, the lack of significant associations for AT thickness indicates that while CSA may respond to elevation changes, thickness may not be influenced in the same manner. A previous study reported the range of motion of the ankle was about 15% greater during uphill running than at level running [34]. As mentioned in the introduction, tendons play a critical role in the stretch–shortening cycle; according to this, the AT participates in this cycle thanks to the plantar and dorsal flexion movements of the ankle. While the ankle dorsal flexion allows the stretching of this tendon while storing elastic energy, the ankle plantar flexion shortens the AT, facilitating the release of said energy and, consequently, the movement of the runner. Therefore, it can be speculated that the accumulated elevation gained during uphill running will provoke greater demands on the AT due to the greater range of motion that occurs in the ankle joint, and these greater demands may explain the tendon morphological differences. In this study, the tendon morphological differences associated with the accumulated elevation gained were significant in the AT but not in the PT. This finding may be explained by the fact that during uphill running, the demands for energy absorption and generation are between two and four times greater in the ankle than in the knee [34]. Therefore, the request for the AT is much greater than that for the PT, which could explain the different responses between them.

The greater demand that trail running provokes on the AT may be one of the reasons why one of the main injuries that occurs in these athletes is Achilles tendinopathy [26]. It is well demonstrated that an excess load, which exceeds the adaptive capacities of the tendon, is the main cause of the development of tendinopathy [35]. Since the results of this study show that tendons adapt to the running training modality (weekly running volume and accumulated elevation gain), trail runners should pay special attention to their tendons’ health by either adapting volume and elevation gain or including proper strength training to their weekly routines in order to optimize tendon health [36,37] and, therefore, optimize performance. However, it is still unknown whether trail runners’ tendon morphology influences biomechanical variables such as stiffness or spatiotemporal parameters, like in road runners [24,25].

Despite the findings reported here, there are some limitations to be considered. First, ultrasound measurements of both PT and AT were performed at a specific location, as described in previous studies [14,25]. Since research on tendon morphology in trail runners is limited, and in view of the significant results found here, it would be interesting to assess whether measurements at distal and proximal sites adapt the same way. Although in this study, trail runners had higher weekly mileage than road runners, the confounding effect of this variable on elevation gain, and vice versa, was controlled for. However, these differences are inherent to their sport, as trail runners typically train and compete over longer distances and with a greater cumulative elevation gain than road runners. Also inherent to trail running is downhill running. Since this has not been considered here, the relationship between PT and AT morphology and downhill running needs to be explored in future studies, as higher peak forces acting on the patellar tendon might promote appropriate adaptations. In this regard, it should also be acknowledged that the amount and type of strength training that athletes perform to complement their running sessions was not controlled. Previous research on strength training habits in runners [38] indicates that lower competitive levels are generally associated with fewer strength training sessions in their programs. Additionally, many runners consider bodyweight exercises, plyometrics, and uphill running as integral components of their strength training routines. Therefore, standardizing the strength training programs followed by each road or trail runner can be challenging, as interpretations of what constitutes strength training may vary. Finally, it should be considered that there were slight differences in the age of the participants between the two groups, with the trail runners being, on average, two years older. However, both groups consisted of runners of competitive age (i.e., 20 to 35 years old), which reinforces the power of the results reported here.

## 5. Conclusions

Trail runners possess larger and thicker AT compared to road runners due to high weekly mileage and accumulated positive elevation gain. This suggests that the mechanical overload experienced by trail runners may induce an adaptive response to training volume and the demands of uphill running. Therefore, examining the tendon morphology of trail runners could provide valuable insights into their adaptation to the specific load of this sport, potentially helping to identify weaknesses and conduct training interventions to improve performance and promote health.

## Figures and Tables

**Table 1:** Demographic description of the participants.

	Road Runners (n = 33)	Trail Runners (n = 33)	*t*-Test (df)	*p*-Value
Age (years) ^	27.9 ± 7.13	30.2 ± 4.52	343.00 (64)	0.010
Height (cm)	172.9 ± 8.44	176.0 ± 8.71	−1.48 (64)	0.144
Weight (kg)	67.7 ± 10.47	66.8 ± 8.77	0.41 (64)	0.683
BMI (kg·m^−2^)	22.5 ± 2.24	21.6 ± 2.50	1.69 (64)	0.096
Weekly volume (km) ^	30 (10)	80 (40)	61.50 (64)	<0.001
Weekly elevation gain (m) ^	50 (30)	2500 (1600)	0.00 (64)	<0.001

BMI: Body Mass Index; ^ Reported as median (IQR). Mann–Whitney U test as Levene’s test is significant (*p* < 0.05), suggesting a violation of the assumption of equal variances.

**Table 2:** Tendon morphology comparisons between road and trail runners.

	Road Runners (n = 33)	Trail Runners (n = 33)	Difference (Mean [95% CI])	*p*-Value
Patellar Tendon				
Thickness (mm)	3.35 ± 0.49	3.47 ± 0.48	0.12 (0.12–0.36)	0.330
CSA (mm^2^)	82.29 (23.26) ^	84.55 ± 17.51	4.91 §	0.516
Thickness normalized (mm kg^−1^)	0.07 (0.01) ^	0.08 ± 0.01	0.01 §	0.203
CSA normalized (mm^2^ kg^−1^)	1.87 ± 0.33	1.91 ± 0.40	0.05 (0.13–0.23)	0.596
Achilles Tendon				
Thickness (mm)	4.93 ± 0.59	5.33 (0.54) ^	0.46 §	<0.001
CSA (mm^2^)	49.61 ± 6.51	66.70 (18.3) ^	17.70 §	<0.001
Thickness normalized (mm kg^−1^)	0.11 ± 0.02	0.12 (0.03) ^	0.01 §	0.002
CSA normalized (mm^2^ kg^−1^)	1.11 ± 0.17	1.59 ± 0.31	0.47 (0.35–0.60)	<0.001

CSA: Cross-sectional area. *p*-values are reported via Student’s *t*. ^ Reported as median (IQR). Mann–Whitney U test as Levene’s test is significant (*p* < 0.05), suggesting a violation of the assumption of equal variances. § Difference in medians due to the non-normal distribution of one of the groups. Here, *p*-values are reported using the Mann–Whitney U test.

**Table 3:** Pearson’s correlation coefficients (r) for tendon morphology vs. weekly mileage and elevation gain.

	Patellar Tendon		Achilles Tendon	
	r	*p*-Value	r	*p*-Value
Kilometers/week				
Thickness	0.195	0.120	0.270	0.029
CSA	0.099 ^	0.433	0.291 ^	0.019
Thickness normalized	0.100 ^	0.430	0.102 ^	0.419
CSA normalized	0.087	0.492	0.319	0.010
Elevation gain/week				
Thickness	−0.152	0.226	0.108 ^	0.391
CSA	−0.139 ^	0.269	0.332 ^	0.007
Thickness normalized	0.028 ^	0.824	0.334 ^	0.007
CSA normalized	−0.102	0.420	0.196	0.118

^ Spearman’s ρ is reported due to a violation of the assumption of equal variances.

## Data Availability

The data presented in this study are available on request from the corresponding author due to ongoing research.

## References

[1] Franken R., Bekhuis H., Tolsma J. (2022). Running Together: How Sports Partners Keep You Running. Front. Sports Act. Living.

[2] Scheer V., Basset P., Giovanelli N., Vernillo G., Millet G.P., Costa R.J.S. (2020). Defining Off-road Running: A Position Statement from the Ultra Sports Science Foundation. Int. J. Sports Med..

[3] Sabater Pastor F., Besson T., Berthet M., Varesco G., Kennouche D., Dandrieux P.E., Rossi J., Millet G.Y. (2023). Elite Road vs. Trail Runners: Comparing Economy, Biomechanics, Strength, and Power. J. Strength Cond. Res..

[4] Gruber M., Kramer A., Mulder E., Rittweger J. (2019). The Importance of Impact Loading and the Stretch Shortening Cycle for Spaceflight Countermeasures. Front. Physiol..

[5] Vogt M., Hoppeler H.H. (2014). Eccentric exercise: Mechanisms and effects when used as training regime or training adjunct. J. Appl. Physiol..

[6] Gottschall J.S., Kram R. (2005). Ground reaction forces during downhill and uphill running. J. Biomech..

[7] Lussiana T., Fabre N., Hebert-Losier K., Mourot L. (2013). Effect of slope and footwear on running economy and kinematics. Scand. J. Med. Sci. Sports.

[8] Rice H., Patel M. (2017). Manipulation of Foot Strike and Footwear Increases Achilles Tendon Loading During Running. Am. J. Sports Med..

[9] Vernillo G., Giandolini M., Edwards W.B., Morin J.B., Samozino P., Horvais N., Millet G.Y. (2017). Biomechanics and Physiology of Uphill and Downhill Running. Sports Med..

[10] Kulmala J.P., Avela J., Pasanen K., Parkkari J. (2013). Forefoot strikers exhibit lower running-induced knee loading than rearfoot strikers. Med. Sci. Sports Exerc..

[11] Xu Y., Yuan P., Wang R., Wang D., Liu J., Zhou H. (2021). Effects of Foot Strike Techniques on Running Biomechanics: A Systematic Review and Meta-analysis. Sports Health.

[12] Devaprakash D., Obst S.J., Lloyd D.G., Barrett R.S., Kennedy B., Ball I., Adams K.L., Collings T.J., Davico G., Hunter A. (2020). The Free Achilles Tendon Is Shorter, Stiffer, Has Larger Cross-Sectional Area and Longer T2(*) Relaxation Time in Trained Middle-Distance Runners Compared to Healthy Controls. Front. Physiol..

[13] Hullfish T.J., Hagan K.L., Casey E., Baxter J.R. (2018). Achilles tendon structure differs between competitive distance runners and nonrunners despite no clinical signs or symptoms of midsubstance tendinopathy. J. Appl. Physiol..

[14] Kubo K., Tabata T., Ikebukuro T., Igarashi K., Yata H., Tsunoda N. (2010). Effects of mechanical properties of muscle and tendon on performance in long distance runners. Eur. J. Appl. Physiol..

[15] Salinero J.J., Lara B., Gutierrez-Hellin J., Gallo-Salazar C., Areces F., Jiménez F., Coso J.D. (2020). Thickness and Cross-Sectional Area of the Achilles Tendon in Marathon Runners: A Cross-Sectional Study. Rev. Bras. Med. Esporte.

[16] Rubio-Peirotén A., García-Pinillos F., Cartón-Llorente A., Jaén-Carrillo D., Abat F., Roche-Seruendo L.E. (2022). Lower-Limb Connective Tissue Morphologic Characteristics in Runners. How Do They Relate with Running Biomechanics and Tendon Pathology? A Systematic Review. Muscles Ligaments Tendons J..

[17] Veronesi F., Borsari V., Contartese D., Xian J., Baldini N., Fini M. (2020). The clinical strategies for tendon repair with biomaterials: A review on rotator cuff and Achilles tendons. J. Biomed. Mater. Res. Part B Appl. Biomater..

[18] Couppé C., Kongsgaard M., Aagaard P., Hansen P., Bojsen-Moller J., Kjaer M., Magnusson S.P. (2008). Habitual loading results in tendon hypertrophy and increased stiffness of the human patellar tendon. J. Appl. Physiol..

[19] Calvo-Rubio M., Garcia-Domiguez E., Tamayo-Torres E., Soto-Rodríguez S., Olaso-Gonzalez G., Ferrucci L., de Cabo R., Gómez-Cabrera M.C. (2024). The repeated bout effect evokes the training-induced skeletal muscle cellular memory. Free. Radic. Biol. Med..

[20] Machado E., Lanferdini F.J., da Silva E.S., Geremia J.M., Sonda F.C., Fletcher J.R., Vaz M.A., Peyré-Tartaruga L.A. (2021). Triceps Surae Muscle-Tendon Properties as Determinants of the Metabolic Cost in Trained Long-Distance Runners. Front. Physiol..

[21] Kovács B., Kóbor I., Gyimes Z., Sebestyén Ö., Tihanyi J. (2020). Lower leg muscle–tendon unit characteristics are related to marathon running performance. Sci. Rep..

[22] Dar G., Waddington G., Stern M., Dotan N., Steinberg N. (2020). Differences Between Long Distance Road Runners and Trail Runners in Achilles Tendon Structure and Jumping and Balance Performance. PM R J. Inj. Funct. Rehabil..

[23] Monte A., Maganaris C., Baltzopoulos V., Zamparo P. (2020). The influence of Achilles tendon mechanical behaviour on “apparent” efficiency during running at different speeds. Eur. J. Appl. Physiol..

[24] Rubio-Peiroten A., Garcia-Pinillos F., Jaen-Carrillo D., Carton-Llorente A., Abat F., Roche-Seruendo L.E. (2021). Relationship between Connective Tissue Morphology and Lower-Limb Stiffness in Endurance Runners. A Prospective Study. Int. J. Environ. Res. Public Health.

[25] Rubio-Peiroten A., Carton-Llorente A., Roche-Seruendo L.E., Jaen-Carrillo D. (2023). Larger Achilles and plantar fascia induce lower duty factor during barefoot running. J. Sci. Med. Sport.

[26] Viljoen C.T., Janse van Rensburg D.C., Verhagen E., van Mechelen W., Tomas R., Schoeman M., Scheepers S., Korkie E. (2021). Epidemiology of Injury and Illness Among Trail Runners: A Systematic Review. Sports Med..

[27] Del Bano-Aledo M.E., Martinez-Paya J.J., Rios-Diaz J., Mejias-Suarez S., Serrano-Carmona S., de Groot-Ferrando A. (2017). Ultrasound measures of tendon thickness: Intra-rater, Inter-rater and Inter-machine reliability. Muscles Ligaments Tendons J..

[28] Cureton E.E. (1956). Rank-biserial correlation. Psychometrika.

[29] Cohen J. (2013). Statistical Power Analysis for the Behavioral Sciences.

[30] Magnusson S.P., Kjaer M. (2003). Region-specific differences in Achilles tendon cross-sectional area in runners and non-runners. Eur. J. Appl. Physiol..

[31] Rosager S., Aagaard P., Dyhre-Poulsen P., Neergaard K., Kjaer M., Magnusson S.P. (2002). Load-displacement properties of the human triceps surae aponeurosis and tendon in runners and non-runners. Scand. J. Med. Sci. Sports.

[32] Kubo K., Ikebukuro T., Yata H., Tomita M., Okada M. (2011). Morphological and mechanical properties of muscle and tendon in highly trained sprinters. J. Appl. Biomech..

[33] Kubo K., Miyazaki D., Ikebukuro T., Yata H., Okada M., Tsunoda N. (2017). Active muscle and tendon stiffness of plantar flexors in sprinters. J. Sports Sci..

[34] Khassetarash A., Vernillo G., Martinez A., Baggaley M., Giandolini M., Horvais N., Millet G.Y., Edwards W.B. (2020). Biomechanics of graded running: Part II-Joint kinematics and kinetics. Scand. J. Med. Sci. Sports.

[35] Cook J.L., Purdam C.R. (2009). Is tendon pathology a continuum? A pathology model to explain the clinical presentation of load-induced tendinopathy. Br. J. Sports Med..

[36] Kubo K., Ikebukuro T., Yata H., Tsunoda N., Kanehisa H. (2010). Time course of changes in muscle and tendon properties during strength training and detraining. J. Strength Cond. Res..

[37] Wiesinger H.-P., Koesters A., Mueller E., Seynnes O.R. (2015). Effects of increased loading on in vivo tendon properties: A systematic review. Med. Sci. Sports Exerc..

[38] García-Pinillos F., Lago-Fuentes C., Jaén-Carrillo D., Bujalance-Moreno P., Latorre-Román P., Roche-Seruendo L.E., Ramirez-Campillo R. (2020). Strength Training Habits in Amateur Endurance Runners in Spain: Influence of Athletic Level. Int. J. Environ. Res. Public Health.

